# An *ex vivo* salivary lubrication system to mimic xerostomic conditions and to predict the lubricating properties of xerostomia relieving agents

**DOI:** 10.1038/s41598-018-27380-7

**Published:** 2018-06-14

**Authors:** Jeroen Vinke, Hans J. Kaper, Arjan Vissink, Prashant K. Sharma

**Affiliations:** 1 University of Groningen and University Medical Center Groningen, Department of Biomedical Engineering, Groningen, The Netherlands; 2 University of Groningen and University Medical Center Groningen, Department of Oral Maxillofacial Surgery, Groningen, The Netherlands

## Abstract

Advances in medical research has resulted in successful treatment of many life-threatening infectious diseases as well as autoimmune and lifestyle-related diseases, increasing life-expectancy of both the developed and developing world. As a result of a growing ageing population, the focus has also turned on chronic diseases which seriously affect the quality of older patient life. Xerostomia (dry mouth) is one such condition, which leads to bad oral health and difficulty in consumption of dry foods and speech. Saliva substitutes are used to ease symptoms. However, they often don’t work properly and objective comparison of saliva substitutes to mimic natural salivary functions does not exist. The study thus aims to develop an *ex vivo* friction assay simulating dry mouth conditions and facilitating objective comparison of saliva substitutes. A reciprocating sliding tongue-enamel system was developed and compared to a PDMS (polydimethylsiloxane)-PDMS friction system. The tongue-enamel system, but not the PDMS-PDMS model, showed high mucin-containing saliva (unstimulated and submandibular/sublingual saliva) to give higher Relief than mucin-poor lubricants (water, parotid saliva, Dentaid Xeros) and correlated well (r = 0.97) with *in vivo* mouth feel. The tongue-enamel friction system mimicked dry mouth conditions and relief and seems suited to test agents meant to lubricate desiccated oral surfaces.

## Introduction

Saliva is the collective secretion from parotid, submandibular, sublingual and minor saliva glands and consists of approximately 99% water with some electrolytes, proteins, glycoproteins and enzymes^1–3^. Proteins and glycoproteins from saliva adsorb on the entire oral surface applying a salivary condition film (SCF). Besides taste perception, digestion, teeth integrity and antimicrobial activity, saliva and the SCF together provide lubrication during oral functioning (speech, chewing, swallowing) where frictionless sliding between different soft and hard tissue surfaces is essential.

Reduced saliva secretion (hyposalivation) and changed saliva composition are associated with xerostomia, i.e. a dry mouth feel^4,5^, and a variety of additional dryness related complaints^6^. Approximately 25% of all elderly and 50–60% of hospitalized elderly suffer from xerostomia^4,7,8^. Major causes are the use of medication^9,10^, Sjögren’s syndrome^5,11^, and radiotherapy in the maxillofacial region^12,13^. Although xerostomia is not a life-threatening condition, it certainly reduces the patient’s quality of life, with a significant negative impact on healthy ageing^14,15^.

A common action that patients can undertake against xerostomia related complaints are maintaining good oral hygiene complemented by regular hydration of the oral cavity using saliva substitutes^16,17^. Saliva substitutes only relieve the symptoms of a dry mouth, although not always effective, instead of being a treatment restoring salivary gland function^18^. Comparison of saliva substitutes for their efficacy to relieve dry mouth remains difficult in absence of reliable *in vitro* or *ex vivo* models which truly mimic the dry mouth condition; this leaves the comparison to be made *in vivo* via the use of a questionnaire^19–21^. However, questionnaires are justly recognized to be valuable in representing the patient’s real experiences of dry mouth relief, it remains difficult to develop new materials or formulations with the help of the questionnaire alone because of the high variability. Ben-Aryeh *et al*. even questioned the reliability of questionnaires to score saliva substitutes since differences in perception exist between patients^4^. Moreover, placebos were found to reveal similar results in relieving symptoms as saliva substitutes which makes it difficult to rate a saliva substitute as truly effective^20,22,23^. This indicates a great need of a simple *ex vivo* test setup where the dry mouth condition can be simulated. This test could then be used to optimize the material and formulation development, although patient questionnaires still should be used as a final step in product development to prove the effectiveness *in vivo*.

In contrast to articular joint lubrication, where friction coefficients between articulating surfaces are very well known^24^, no *in vivo* measurements for oral friction coefficients exist. At most, there are some *in vitro* estimates. In the past, a rotating polydimethylsiloxane (PDMS) ball or disk sliding on a PDMS disk has been a popular setup to mimic lubrication properties of saliva on soft tissues^25–27^. This setup shows a 100-fold drop in the coefficient of friction (COF) from ≈2.5 in dry condition to ≈0.025 when the test surface is lubricated with whole saliva^25^. Such low friction coefficients are similar to well lubricated artificial knee joint friction^24^ and thus seem appealing at first sight. However, salivary lubrication of oral surfaces is radically different from articular joint because the articular joints require perception free lubrication, whereas in the oral cavity a certain level of perception is essential, e.g., to feel the texture of food or to feel the smoothness of the tooth surface after plaque removal. Differences in measured friction coefficients are essential to perceive the difference between two different surfaces or textures^28,29^. Differences can be easily sensed if the absolute COF value is relatively high, as is needed for tactile tribology^30^. Thus, it may not be surprising if the *in vivo* salivary friction coefficient is higher than that in articular joints and the ones measured when applying the PDMS system. In a separate study, a COF of 0.25 ± 0.03 has been reported between a stainless steel ball sliding against a saliva coated porcine tongue^31^, which supports the premise of relatively high COF in the oral cavity. This also strongly suggests the use of biological surfaces in the development of a system to mimic dry mouth conditions instead of artificial PDMS surfaces. To mimic *in vivo* situations even more closely, it is preferred to use tooth enamel, instead of stainless steel, for sliding against the porcine tongue. Material properties play a role in this, since protein adsorption could be different in stainless-steel surfaces as in enamel. Therefore, the aim of this study was to develop an *ex vivo*, tongue-enamel reciprocating sliding friction system that can mimic dry mouth conditions and in which the efficacy of saliva substitutes can be compared to that of natural saliva. The tongue-enamel system was compared to a PDMS-PDMS system for their ability to differentiate between water, stimulated and unstimulated saliva, and saliva from different glandular sources.

## Methods

### Saliva collection and preparation

Standard protocols were followed during the collection and preparation of unstimulated (UWS), stimulated (SWS), reconstituted (RWS) whole saliva, and glandular (parotid (PS) and submandibular/sublingual (SMSL)) saliva^32^, which are described in detail below. Two brands of commercially available mouth lubricants were used in this study: Saliva Orthana (SO), (Pharmachemie BV, Haarlem, The Netherlands), and Dentaid Xeros mouthwash (DX), (Dentaid SL, Barcelona, Spain). Demineralized water (DW) is used as a control.

Whole saliva was donated voluntarily by 5 healthy adult donors (age 28.2 ± 2.8 years) of both genders (3 males, 2 females) recruited from the department of Biomedical Engineering of the University Medical Centre Groningen, the Netherlands. All methods were performed in accordance with the relevant guidelines and regulations under the approval of the Medical Ethics Review Board of the University Medical Center Groningen (approval no. M17.217043, M09.069162 and UMCG IRB #2008109) and donors gave their informed consent. The experiments were done in triplicate and therefore, collecting of whole saliva was done on the same day of the week and at 10:00 a.m. for three weeks. Participants did not eat and drink for one hour in advance. Before collecting any saliva, the mouth was rinsed well with tap water.

#### Unstimulated human whole saliva

For acquiring UWS the drooling technique was used. With minimal oral movements, donors collected saliva in their mouth for 5 minutes. The produced saliva was collected in containers. From the collected saliva of each donor, an amount of 100 µL of saliva was pooled with that of all other donors, stored on ice and was used immediately in experiments.

#### Stimulated human whole saliva (SWS)

From the same 5 donors, mechanically stimulated (chewing on Parafilm) saliva was collected for 15 minutes, 5 minutes after the UWS collection. Meanwhile, participants were allowed to expectorate saliva in ice cooled sterile flasks. The SWS was pooled and clarified by centrifugation at 10000 rpm (avg. 10100 g) at 10 °C for 5 minutes. For stabilizing the SWS, the protease enzymes were inhibited by adding phenylmethylsulfonyl fluoride (0.2 M in EtOH) (final concentration of 1 mM) and centrifuged again (same settings). 1 mL of the supernatant saliva was stored on ice for experimental use the same day. Abundant SWS was dialyzed in a graduate cylinder and stirred overnight at 4 °C against demineralized water. Part of this saliva was freeze-dried for storage and for making RWS as described below.

For the mouth feel survey, a group of 22 healthy volunteers (8 males, 14 females) (18–21 years) donated SWS as described above.

#### Reconstituted human whole saliva

For acquiring pooled RWS, 15 mg of the previous freeze-dried SWS was dissolved in adhesion buffer (10% 0.5 M KCl; 0.2% 0.5 M KPi; 0.1% 0.5 M CaCl_2_; 89.7% demineralized water) at a concentration of 1.5 mg/ml and stirred at low velocity for 30 minutes. The saliva was centrifuged at 10000 rpm (avg. 10100 g) at 10 °C for 5 minutes. The supernatant was stored in ice cooled tube till used and called RWS.

#### Glandular saliva

Glandular saliva was collected from 1 healthy male donor (age 24). Parotid saliva was collected by the use of Lashley cups and a vacuum pump. The mucosa was dried with gauze before the Lashley cups were placed and kept over the parotid duct openings using a maximum pump suction of 1 bar. Parotid saliva flow was passive i.e. no stimulatory products were used to stimulate saliva secretion. Meanwhile secreted submandibular/sublingual saliva was collected by syringe aspiration. The collected saliva was stored separately in ice cooled tubes.

### Preparation of the tongue-enamel friction pair

**Figure 1:**
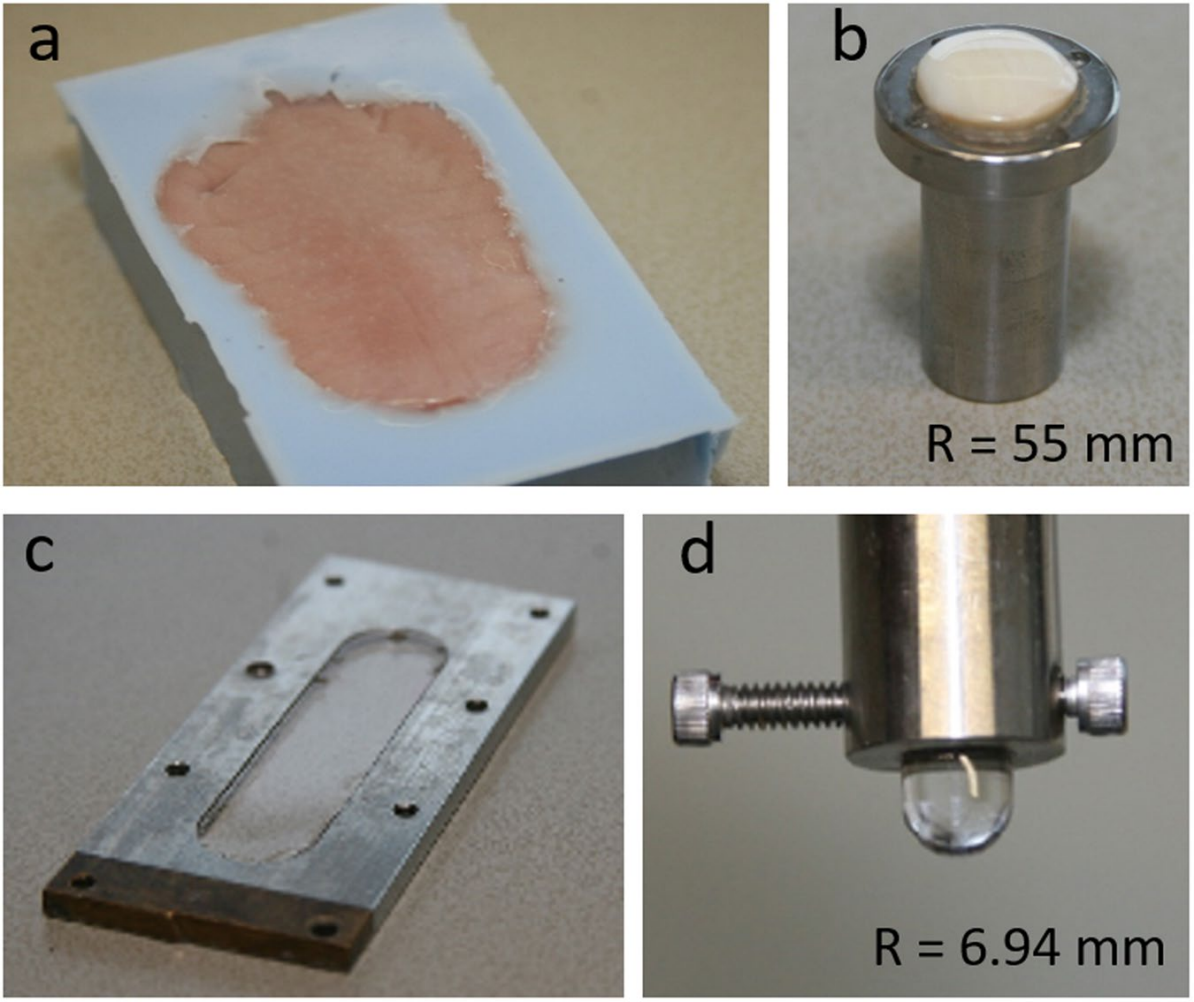
The tongue-enamel and PDMS-PDMS surfaces. (**a**) The porcine tongue embedded in the duplicating silicone. (**b**) The rounded and polished piece of enamel with a radius of curvature of 55 mm fixated in a stainless-steel holder. (**c**) The stainless-steel flow cell with in the middle the PDMS flat surface. (**d**) The PDMS pin with a radius of curvature of 3 mm was fixated in a stainless-steel holder that was fitted to the load cell of the testing device.

Fresh porcine tongues (90 kg, 6 months old pigs) (Kroon BV, Groningen, Netherlands) were rinsed with tap water to wash off coarse waste and put in a bath of ice water for ten minutes, then rinsed with demineralized water for 15 seconds. Excess water was removed by dabbing the tongue with paper towel and tongues were wrapped in cling film. To achieve a horizontal area of the tongues, the wrapped tongues were softly pressed against a glass plate inside a square box by a handmade setup. The box was filled with duplicating silicone, (Fig. 1a) and hardened for 20 minutes. To achieve a similar hydration state of all tongues, the tongues were submerged in adhesion buffer for 30 minutes after opening the cling film and rinsing with demineralized water for 15 s. The tongues were rinsed with demineralized water for 15 seconds again and dap dried (30 times) with precision wipes before experiments. The handling of the tongue could remove any kind of salivary proteins which may exist on it, although no treatment was done to intentionally remove adsorbed proteins.

The enamel used is of bovine origin and is primarily composed of hydroxyapatite, a crystalline calcium phosphate. Bovine teeth were shaped to fit in a stainless-steel holder by grinding its sides with a coarse sandpaper while keeping the anterior side of the tooth intact. The teeth were glued to the stainless-steel holder from the posterior side (Fig. 1b) and stored overnight at room temperature. The enamel was slightly ground in a bench pillar drill aligned above a sphere-shaped diamond grinder with a diameter of 55 mm while being continuously cooled with demineralized water to smoothen the surface. Coarse polishing was performed with a rubber polishing wheel. The final surface finish was obtained by sliding the enamel against a wetted polishing cloth with 0.05-micron alumina micro-polish. The enamel was rinsed with demineralized water and sonicated for 5 minutes to remove any alumina particles and stored in the fridge when not used. The final polishing and sonication were also done after each experiment to remove proteins and to generate a fresh layer of enamel.

### Preparation of the PDMS-PDMS friction pair

PDMS SYLGARD 184 silicone elastomer kit was used to make the PDMS pins and plate. The pins were made by pouring the PDMS in polystyrene U-bottom 96-well plates, and the plates were made upon a glass slide mounted in a flow cell (volume 3 mL), which was filled till the edge with PDMS (Fig. 1c). The PDMS plate and pins were put in a vacuum furnace at room temp and a vacuum of 200 torr using a vacuum pump to deaerate. Air bubbles were cleared by reflating the furnace several times. The PDMS pins and plate were incubated overnight at 80 °C and 200 torr pressure to allow crosslinking and were cooled down before experiments. The pins were fixated in a stainless-steel holder to fit in the load cell of the testing device (Fig. 1d).

### Tribology experiments

**Figure 2:**
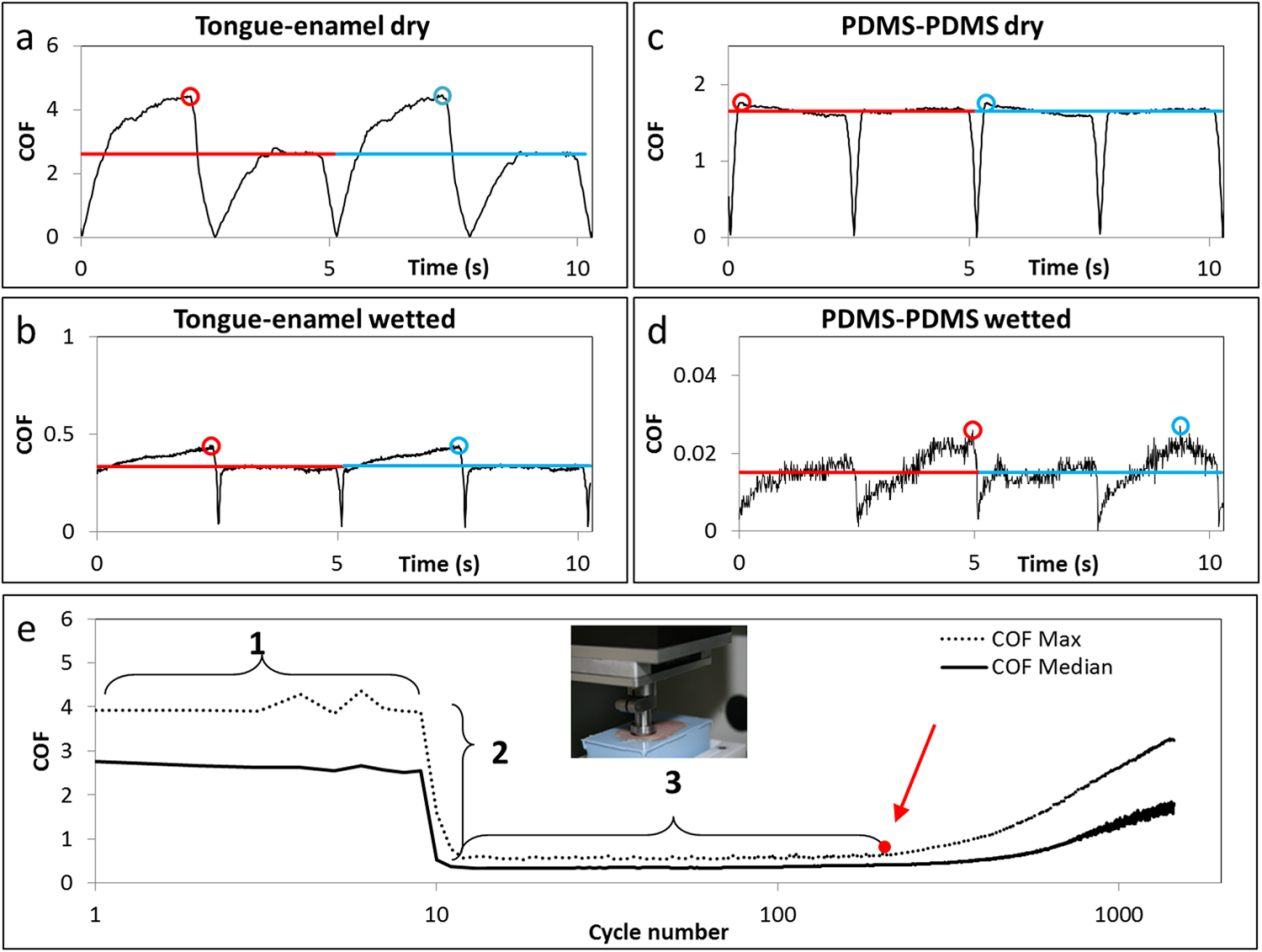
Raw friction profiles of both interacting surfaces and parameter explanation. The raw friction profile of 2 cycles of enamel sliding over the tongue (**a**) in dry condition and (**b**) in lubricated condition. The raw friction profile of 2 cycles of the PDMS pin sliding over the PDMS surface (**c**) in dry condition and (**d**) in lubricated condition. The red and blue circles depict the maximum COF of the two consecutive cycles in all graphs a–d, whereas the red and blue lines correspond to the calculated median value of each cycle. Here, one can see that the median value corresponds to the plateau value. (**e**) The median and maximum friction of every cycle are plotted first during dry sliding (1) after hydration with 20 µL of lubricant (2). Point 2 represents the relief which the patient will feel immediately after rinsing the mouth with the particular lubricant and region 3 represents the duration for which patients feel relieved of the signs of dry mouth. Experiments were done on a UMT-3 device (inset of e).

The prepared tongues were immobilized on top of the lower drive in the universal mechanical tester (UMT-3, CETR Inc., USA) (Fig. 2e inset). The steel holder with the enamel was inserted in the load cell (range 0.1–10 N). The enamel was set above a flat spot of the tongue before running consecutive cycles in back- and forward direction. The applied vertical force of the enamel on the tongue was experimentally determined at 0.25 N data and protocol are shown in the Supplementary information (Supplementary Table S1)^33^. The sliding velocity was set at 4 mm/s over a sliding distance of 10 mm^25,34^.

In the experiments, the UMT-3-device measured the raw COF every 0.01 seconds during all dry and lubricated cycles (Fig. 2a–d). From this raw data, the median and maximum COF (*COF*_*median*_ and *COF*_*max*_) from each cycle was taken for further data analysis. Since we are using increase in friction as a sign of dry mouth, the *COF*_*max*_ is presumed to be the first indication of drying mouth felt by the patient and urges the patient to wet their mouths. The median cycle values correspond to the plateau values from the cycles as depicted in Fig. 2a–d. Also the median friction gives better insights in the process of drying out since medians are less sensitive for outliers than maximums. Thus, both the maximum and median friction of all cycles were plotted as a function of time (cycle number), (Fig. 2e).

### Mimicking dry mouth surfaces

Each experiment was performed by following a set of steps (Fig. 2e). In step 1, the enamel was slid against the tongue for 10 cycles in dry condition, i.e., fully hydrated tongue but in absence of any free water. Stabilized COF in this step acts as dry baseline. In step 2, the sliding was stopped and a drop of 20 µL of lubricant was placed at the tongue-enamel interface. The sliding started and a quick drop in COF should be observed. The fold drop in *COF*_*median*_ and *COF*_*max*_ were designated as ‘Relief’ as per equations (1) and (2). The ratio was taken because often order of magnitude differences could be observed in the values of *COF*.

1 $$Relie{f}_{from-median}=CO{F}_{median,average}^{Step1}/CO{F}_{median}^{Step\,2}$$

2 $$Relie{f}_{from-max}=CO{F}_{max,average}^{Step1}/CO{F}_{max}^{Step\,2}$$

To overcome tongue-specific deviation an overall average of both the medians (*COF*_*median, average*_) and the maximums (*COF*_*max, average*_) from step 1 of all performed experiments were taken to calculate the relief from. Both *COF*_*median*_ and *COF*_*max*_ were continuously monitored during step 3 (Fig. 2e), after some time the two COFs should start to rise. The duration for which the COF remains low is designated to as ‘Relief period’. The end of the Relief period was taken as the point, either on maximum or median COF, where a large change in slope (red spot in Fig. 2e) was observed. We hypothesize that saliva from different sources can be compared based on these two parameters i.e. Relief and Relief period (both based on *COF*_*median*_ and *COF*_*max*_).

For PDMS experiments, the PDMS plate was mounted upon the lower drive and a PDMS pin was mounted to the load cell. The protocol for mimicking dry mouth conditions was the same as for tongue-enamel interface.

### Mouth-feel survey

For the mouth feel survey, 22 healthy medicine students participated by giving their informed consent and the survey was performed under approval no. M17.217043 from the Medical ethics committee of University Medical Center Groningen. The volunteers completed a questionnaire, which contained four questions (Table 1a). The questions were answered twice, viz., to judge the existing mouth feel after rinsing the mouth with tap water, and immediately after keeping either red wine (RW) or pear juice (PJ) in the mouth for 1 minute. The protocol for PJ and RW preparation is presented in the supplementary information.

**Table 1:** Mouth feel survey.

	Question asked			Range of scores possible			
Q1	How badly do you want to drink?			1–5, 1 immediately, 3 normal, and 5 no need			
Q2	How smooth are your teeth?			1–5, 1 very rough, 3 normal and 5 very smooth			
Q3	How smooth is your mucosa?			1–5, 1 very rough, 3 normal and 5 very smooth			
Q4	How dry is your mouth?			1–5, 1 extremely dry, 3 normal and 5 extremely wet			
Rinsing with	n	Q1	Q2	Q3	Q4	Relief Tongue-enamel system	Relief PDMS-PDMS system
Tap water	84	3.27 ± 0.45	3.31 ± 0.43	3.25 ± 0.30	3.10 ± 0.28	2.70 ± 0.43	91.54 ± 9.10
Red wine	42	1.69 ± 0.41	2.16 ± 0.38	2.20 ± 0.54	1.98 ± 0.35	1.77 ± 0.37	59.30 ± 8.40*
Pear juice	42	4.31 ± 0.53	3.75 ± 0.53	3.90 ± 0.40	3.97 ± 0.34	3.09 ± 0.43	60.11 ± 6.39*

A table presenting the list of questions asked to healthy volunteers to judge their mouth feel (a) and a table presenting the mouth feel scores for 4 questions immediately after rinsing the mouth with water, red wine (RW) and pear juice (PJ) for 1 minute (b). For the survey questions, comparison between pear juice and red wine was made using a paired t-test. The table also shows the *ex vivo* Relief obtained by saliva diluted with DW, RW and PJ for the two friction systems.

Taking a sip of PJ or RW and keeping it in the mouth for 1 minute stimulates saliva production resulting in a blend of PJ or RW with saliva^35^. Thus, we set the extent of dilution arbitrary to 1:1 to mimic the *in vivo* condition *ex vivo*^36^. The *in vivo* data were compared with *ex vivo* measurements of friction between the tongue and enamel and PDMS on PDMS lubricated with 20 µL of a mixture of SWS with PJ (SWS + PJ), RW (SWS + RW) and DW (SWS + DW) in a 1:1 ratio. The comparison of models could only be descriptive, because there is no statistical analysis possible for comparing these kinds of *in vivo* and *ex vivo* data. The saliva used in these *ex vivo* measurements was pooled saliva obtained from the same volunteers who took part in the survey.

### Statistics

The variability in the average values was reported in terms of standard deviation (SD). One-way analysis of variances (ANOVA) was used to compare different groups, followed by Bonferroni post-hoc tests to compare between different groups using GraphPad Prism (5.00). Paired Student’s t-tests were performed unless it was clearly mentioned. For the correlation assessment, the Pearson’s correlation coefficient ‘r’ was used.

### Data availability

Raw data are readily available.

## Results

### *Ex vivo*

#### Tongue-enamel sliding system

Since each experiment started with step 1 (Fig. 2e), i.e., tongue-enamel dry sliding, a large data set of 148 experiments was available with an average *COF*_*max*_ and *COF*_*median*_ of 3.21 ± 1.53 and 1.87 ± 0.75, respectively, which represent ${{COF}}_{{\max },{average}}^{{Step}1}$ and ${{COF}}_{{median},{average}}^{{Step}1}$ from equation (1) and (2). These numbers represent the baseline of which the ‘Relief’ of the natural saliva and saliva substitutes were calculated. Dry *COF*_*max*_ was significantly higher than *COF*_*median*_ (p < 0.0001).

**Figure 3:**
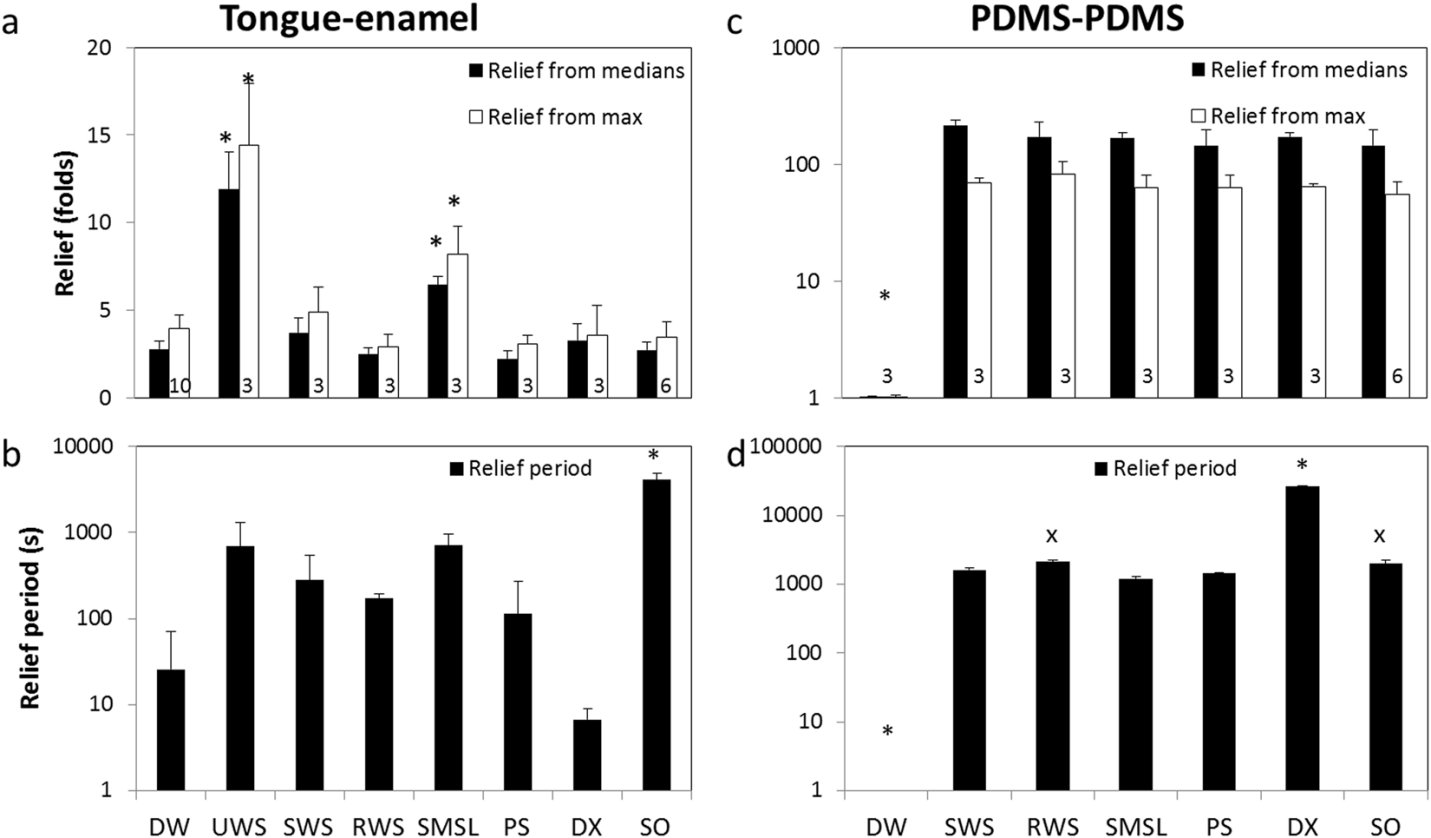
System comparison by parameters. The dry mouth parameters calculated based on tongue-enamel (**a**,**b**) and PDMS-PDMS (**c**,**d**) sliding friction in presence of natural saliva and saliva substitutes. * represents statistical differences compared to all others, and ^x^ to SMSL, with a significance level of 5%. The average and standard deviation were calculated from the number of experiments mentioned in the column in (**a**) and (**c**). (**a**) Relief calculated based on both *COF*_*median*_ and *COF*_*max*_ between tongue and enamel. (**b**) The Relief period in seconds taking both the *COF*_*median*_ and *COF*_*max*_ into consideration for all lubricants. (**c**) Relief based on both *COF*_*median*_ and *COF*_*max*_ for PDMS-PDMS. (**d**) The Relief period for PDMS-PDMS in seconds taking both the *COF*_*median*_ and *COF*_*max*_ into consideration for all lubricants.

DW (n = 10) provided a *Relief*_*from max*_ of 3.97 ± 0.73 (Fig. 3a, p < 0.001). UWS and SMSL provided a much higher Relief than DW and most other lubricants (Fig. 3a). SMSL and UWS resulted in a high Relief, i.e., >8 -fold decrease in *COF*_*max*_ whereas SWS, RWS, PS, SO and DX provide low Relief, i.e., a three- to six-fold decrease in *COF*_*max*_, which are very similar to the Relief of DW.

Figure 3b shows the ‘Relief period’ of either human saliva or saliva substitutes in seconds on the tongue-enamel sliding system. DW provided Relief for 25 s. DX resulted in an even a smaller Relief period (7 ± 2 s), whereas SWS, RWS and PS resulted in a slightly longer Relief period (278, 171 and 113 s, respectively). The UWS and SMSL provided Relief for much longer periods (692 and 708 s, respectively) whereas SO resulted in a very long-lasting Relief period (68 minutes), which was significantly higher from all others (p < 0.05).

### PDMS-PDMS sliding system

The average *COF*_*max*_ and *COF*_*median*_ during dry PDMS-PDMS sliding from 33 experiments were 1.84 ± 0.088 and 1.71 ± 0.056, respectively, (p < 0.0001). Again, these values represent ${{COF}}_{{\max },{average}}^{{Step}1}$ and ${{COF}}_{{median},{average}}^{{Step}1}$ from equations (1) and (2) as the baseline of which the Reliefs were calculated. The *COF*_*max*_ value of the PDMS-PDMS sliding was significantly lower than the one obtained from dry tongue-enamel sliding (p < 0.001, unpaired Student’s t-test), but *COF*_*median*_ did not differ (p > 0.05, unpaired Student’s t-test). Upon the introduction of DW at the PDMS-PDMS interface, no decrease in friction was observed. Interestingly, all the lubricants showed between a 55- and an 82-fold *Relief*_*from max*_ and between a 143- and a 216-fold *Relief*_*from median*_ (p < 0.05) (Fig. 3c).

On the PDMS-PDMS sliding system no Relief period was observed for DW whereas DX gave the highest Relief period (465 minutes). SO and RWS gave a relief for about 34 minutes and the other types of saliva gave a relief for a period of 20 to 26 minutes with lowest being SMSL, which was significant lower than RWS and SO (Fig. 3d).

Both the Relief and Relief Period observed on the PDMS-PDMS sliding system was one or two orders of magnitude higher than on the tongue-enamel sliding system.

### Effect of salivary dilution on *ex vivo* Friction

The effect of saliva dilution with water, RW and PJ on the friction parameters (Relief and Relief period) measured *ex vivo* are presented in Supplementary Figure S1. The Relief_from max_ from both the tongue-enamel and PDMS-PDMS friction systems is shown in Table 1b.

### *In vivo*

#### Mouth-feel survey

In Table 1b, the mean scores from 22 healthy volunteers are depicted. For Q1, the need to drink responses correspond with: ‘normal’ for tap water, ‘immediately’ for RW and ‘no need’ for PJ. For Q2, the teeth smoothness is scored as ‘normal’ for tap water, ‘rough’ for RW and ‘smooth’ for PJ; for Q3, the mucosa smoothness is scored as ‘normal’ for tap water, ‘normal’ for RW and ‘smooth’ for PJ; and for Q4, the mouth dryness was scored as ‘normal’ for tap water, ‘dry’ for RW and ‘wet’ for PJ.

### Correlation between the *ex vivo* and *in vivo* results

**Figure 4:**
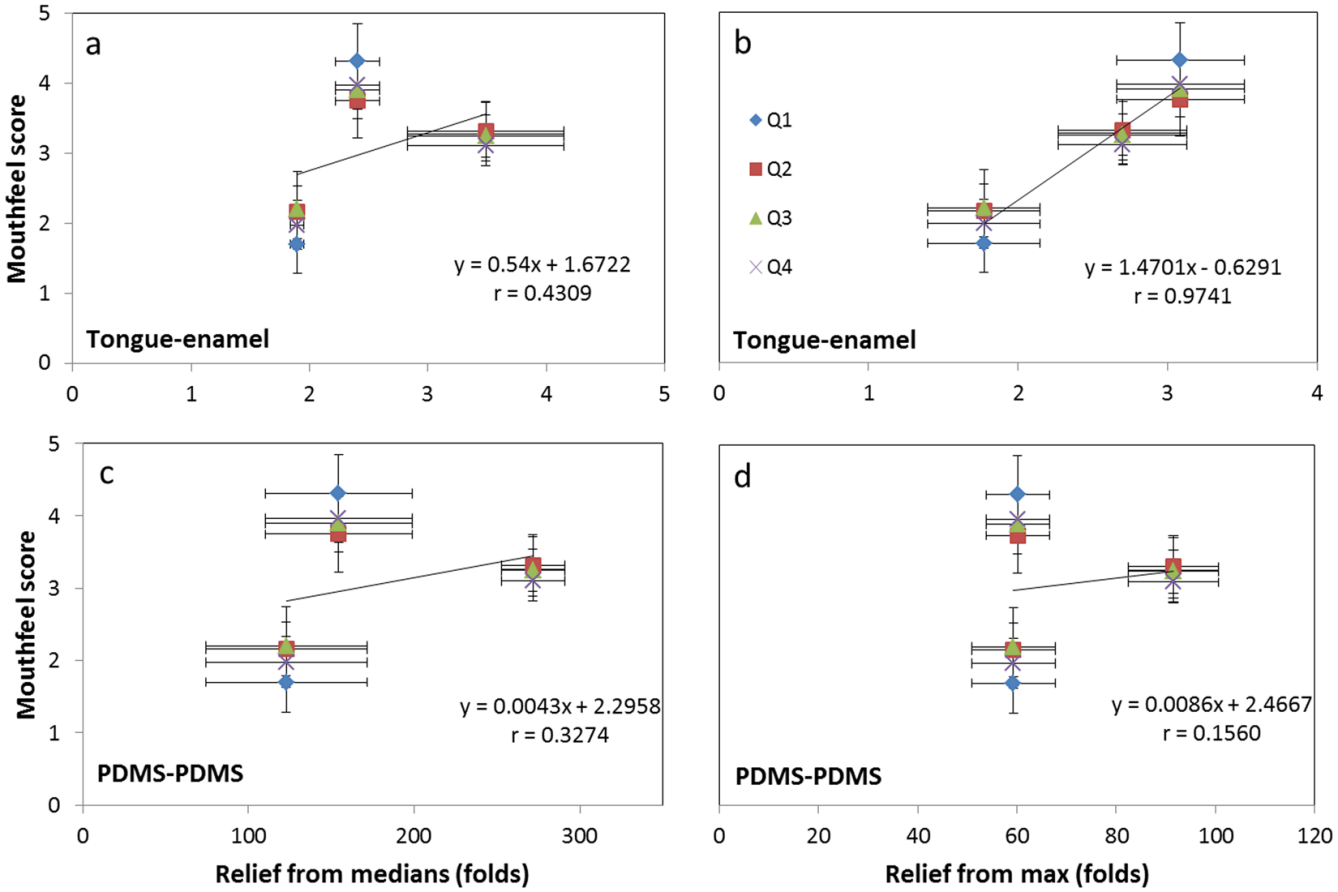
Correlation assessment *in vivo* and *ex vivo* scores. Correlation between mouth-feel survey-scores and the three saliva mixtures’ median and max Relief in the tongue-enamel friction system (**a** and **b**) and PDMS-PDMS friction system (**c** and **d**). Please note that the x-axis’ scales are different. Q1, Q2, Q3 and Q4 represent the questions of the mouth feel survey (Table 1a). The numbers in the columns represent the amount of experiments.

If the Reliefs obtained for saliva mixtures are related to the mouth feel scores (Fig. 4a–d), then we can see that in the Pearson’s correlation coefficients ‘r’. For the tongue-enamel system, the r-values of *Relief*_*from medians*_ and *Relief*_*from max*_ to the mouth feel survey are 0.43 and 0.97 respectively. Correlation between the Relief values from the PDMS-PDMS system and the values from the mouth feel survey showed r-values of 0.33 and 0.16 respectively.

## Discussion

Both the tongue-enamel and PDMS-PDMS friction systems were compared for their suitability to mimic dry mouth conditions by determining parameters like Relief and Relief period for water, SWS, UWS, RWS and glandular saliva. In addition, the Relief observed with both systems was related to the *in vivo* mouth feel. Water is easily accessible and often used by the dry mouth patients to moisten their oral cavity, but in clinical studies this relief only lasts for minutes^18,37^. This phenomenon is explained through poor *in vivo* wettability of oral mucosa and enamel by water^38^. The system developed in this study, tongue-enamel friction, confirmed this phenomenon by showing a Relief of only 4 by adding water (DW) for about 25 s (Fig. 3a,b). Due to the hydrophobicity of PDMS, DW did not show any Relief or Relief period on the PDMS-PDMS system (Fig. 3c,d), which is a clear shortcoming of that friction system.

Mucins are large and highly glycosylated proteins, which contribute to the lubricating properties of saliva^38–41^. Mucins are secreted by the sublingual (SL), submandibular (SM) and minor glands and predominantly absent from parotid saliva (PS)^1,42^. Predominance of mucins in SL and SM gland secretions is reflected by the approximately threefold higher Relief and a six-fold longer Relief period by SMSL on the tongue-enamel friction system as compared to PS. Results obtained on the PDMS-PDMS system were unable to elucidate the physiological difference between types of human saliva.

UWS is mainly composed of submandibular saliva (65–70%) with smaller contributions of parotid (20%) and sublingual saliva (7–8%)^1,2^. Chewing stimulated saliva is mainly composed of parotid saliva (60%) and low amounts of submandibular saliva^43,44^. Predominance of SMSL and thus mucins in UWS causes UWS to provide about a twofold (2.1) higher Relief and a 2.5-fold longer Relief period as compared to SWS on the tongue-enamel friction system. These findings are in line with the findings from Prinz *et al*., who found better lubrication of UWS compared to SWS in continuous friction between tongue and oesophagus mucosa^33^. Differences in Relief and Relief period between SWS and RWS are also an indication of lower content of the lubricating glycoproteins in RWS, probably caused by the processing of RWS which has been shown to impact its composition^45,46^. Although not significant, it is striking to see that in both Relief and Relief period, a similar trend could be seen between the whole and the glandular salivas.

Notable is the poor Relief provided by SO. Due to the mucin content (3% porcine gastric mucin^37^) in SO one could expect a higher Relief compared to mucin-poor saliva and DX. However, as Madsen *et al*. reported, in thin film lubrication, submandibular mucin coating resulted in a much lower friction coefficient as compared to gastric mucin^47^. This can also explain why UWS provided a significantly better Relief than SO. The 700-fold longer Relief period of SO, as compared to DX does underline that the choice of lubricative substances matter for the application in saliva substitutes. The 68 minutes Relief period by SO seems to be long compared to clinical study results where the retention time was 20 to 30 minutes^37,48^. This difference could arise because the tongue-enamel system is not subjected to swallowing or interference from other oral surfaces or movements. The PDMS-PDMS friction system showed a 1.2-fold higher Relief and a 13-fold higher Relief period for DX as compared to SO, indicating a different action of work on both system interfaces. The relief period of 7.5 hours for DX on PDMS-PDMS could be due to formation of an oily lubricating film on PDMS even when the water has completely dried, and thereby, clinically irrelevant. One of the ingredients of DX is PEG-40 hydrogenated castor oil, which in fact is a surfactant. Its oily compound would attach very well on the hydrophobic PDMS surfaces and interact with other active ingredient like hydroxyethyl cellulose during sliding to give rise to a highly lubricating, non-aqueous tribofilm. This would lead to much smaller DX Relief periods on tongue compared to PDMS, since the tongue surface is less hydrophobic and can also adsorb some molecules. Clinically no reports have yet been found where DX has been mentioned to give such long periods of relief to the dry mouth patients as shown by the PDMS-PDMS system. In presence of carboxymethyl cellulose (a very similar molecule to DX’ main component hydroxyethyl cellulose), a clinical retention time of only 10–26 minutes was reported^37,48^.

**Figure 5:**
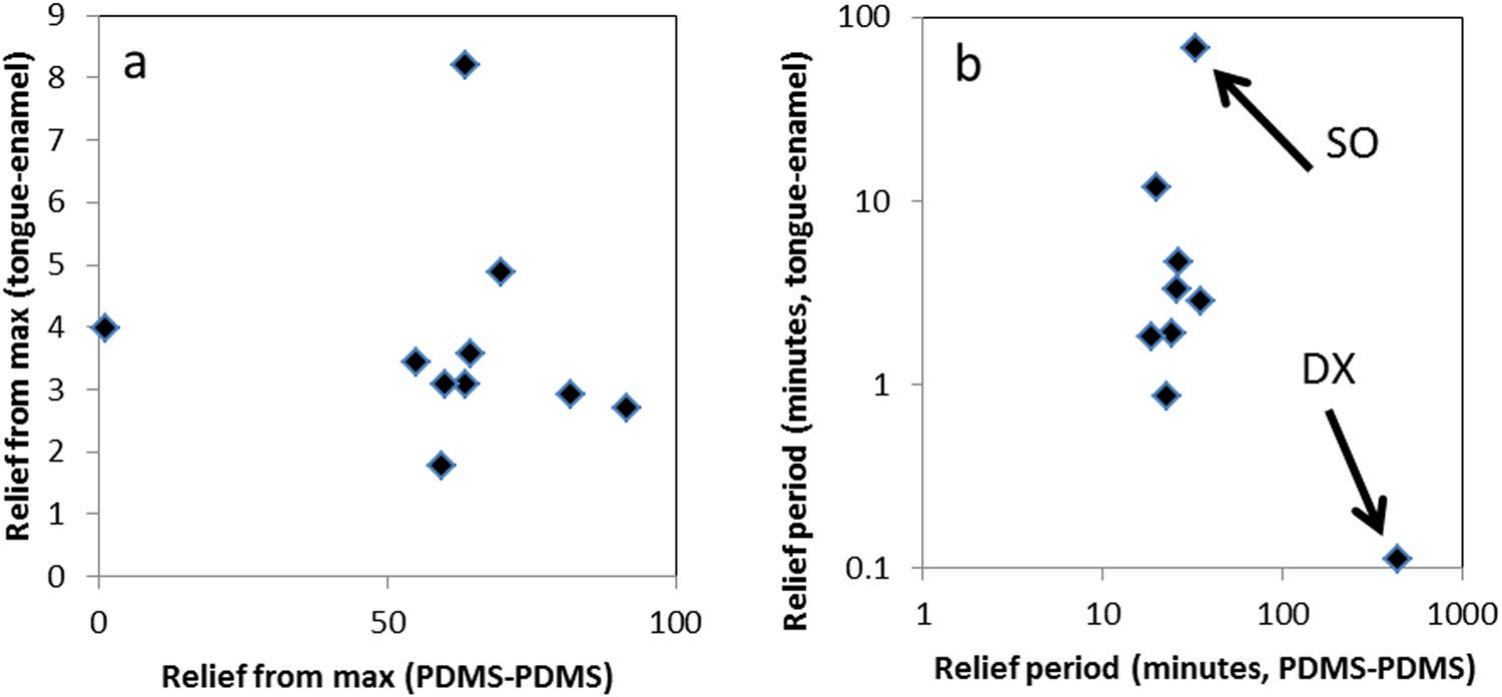
Correlation assessment between the two systems for the tested natural saliva, saliva mixtures and saliva substitutes. Relief: the outlier on the left is water, which showed no relief on the PDMS system, while reliefs the tongue in the tongue-enamel system by a factor of four. The correlation assessment showed no correlation (r of −0.07) (**a**). Relief period: both axes are in log-scale, making the rightmost spot an extreme outlier for the relief period in the PDMS-PDMS system, while SO is the lubricant having the longest relief duration in the tongue-enamel system. Water showed also no Relief period in the PDMS-PDMS system and is off-scale; it showed a short relief period of 25 seconds in the tongue-enamel system. The total correlation assessment delivered an r of 0.13 (**b**).

If the Relief and Relief periods for different human saliva, saliva substitutes and saliva mixtures obtained on the tongue-enamel and the PDMS-PDMS friction system are compared (Fig. 5a,b), then we clearly observe that both systems provide rather dissimilar results. The Relief provided on the PDMS-PDMS system was a 10 to 100-fold higher than shown on the tongue-enamel system, which relates to the clinical situation even less. The COF measured after lubrication ranged from 0.007–0.035, which is within the friction coefficient range in synovial joints^24,49^ and in molar contacts^41^, but not for soft surface contacts. For the Relief period, we see a clear spread of points on the tongue-enamel system whereas on the PDMS-PDMS system, most of the points group together. The only outlier is DX; this indicates that the tongue-enamel friction system is more sensitive in differentiating smaller differences. Furthermore, the outlier DX on PDMS-PDMS is inexplicable. These results indicate that the tongue-enamel system gives clinically more reasonable data than the PDMS-PDMS system, furthermore the results from the tongue-enamel system better correlate with mouth feel (Fig. 4). Relief determined from the tongue-enamel friction system seems to be a much better predictor (especially looking at the Relief_from max_) of the actual mouth feel (Fig. 4) whereas the PDMS-PDMS system does not have this power. Straight line correlations for 3 experimental points may not be completely justified, but the fact is that for all survey questions the trend is similar. The tongue-enamel friction system relates better with actual mouth feel than the PDMS-PDMS friction system. Our findings are in line with other studies which have shown that astringent components induce dry mouth feel as well as elevate friction in tribology experiments^50^.

Based on the obtained results and the above discussions we conclude that the tongue-enamel friction system is better suited to mimic xerostomic conditions and intra-oral friction than the PDMS-PDMS system. The tongue-enamel friction system is sensitive to hydration with water, in contradiction to the PDMS-PDMS system. The tongue-enamel friction system can also distinguish between different compositions and origins of saliva, giving different Relief and Relief period with compositional changes. Mucin-rich saliva delivered especially good results in Relief, and a mucin-containing saliva substitute delivered a long-term relief period necessary in the absence of a continuous flow of natural saliva.

## Electronic supplementary material

Supplementary Data

## References

[1] Humphrey SP, Williamson RT (2001). A review of saliva: normal composition, flow, and function. J. Prosthet. Dent..

[2] de Almeida DV (2008). L. Saliva composition and functions: a comprehensive review. J. Contemp. Dent. Pract..

[3] Dawes C (2015). The functions of human saliva: A review sponsored by the World Workshop on Oral Medicine VI. Arch. Oral Biol..

[4] Ben-Aryeh H, Miron D, Berdicevski I, Szargel R, Gutman D (1985). Xerostomia in the elderly: prevalence, diagnosis, complications and treatment. Gerodontology.

[5] Von Bültzingslöwen I (2007). Salivary dysfunction associated with systemic diseases: systematic review and clinical management recommendations. Oral Surg. Oral Med. Oral Pathol. Oral Radiol. Endod..

[6] Plemons JM, Al-Hashimi I, Marek CL (2014). Managing xerostomia and salivary gland hypofunction. J. Am. Dent. Assoc..

[7] Pajukoski H, Meurman JH, Halonen P, Sulkava R (2001). Prevalence of subjective dry mouth and burning mouth in hospitalized elderly patients and outpatients in relation to saliva, medication, and systemic diseases. *Oral Surgery, Oral Med*. Oral Pathol. Oral Radiol. Endodontology.

[8] Smidt D, Torpet LA, Nauntofte B, Heegaard KM, Pedersen AML (2010). Associations between labial and whole salivary flow rates, systemic diseases and medications in a sample of older people. Community Dent. Oral Epidemiol..

[9] Porter SR, Scully C, Hegarty AM (2004). An update of the etiology and management of xerostomia. *Oral Surgery, Oral Med*. Oral Pathol. Oral Radiol. Endodontology.

[10] Aliko A (2015). World Workshop on Oral Medicine VI: Clinical implications of medication-induced salivary gland dysfunction. Oral Surg. Oral Med. Oral Pathol. Oral Radiol..

[11] Peri Y, Agmon-Levin N, Theodor E, Shoenfeld Y (2012). Sjögren’s syndrome, the old and the new. Best Pract. Res. Clin. Rheumatol..

[12] Vissink A (2010). Clinical management of salivary gland hypofunction and xerostomia in head-and-neck cancer patients: successes and barriers. Int. J. Radiat. Oncol. Biol. Phys..

[13] Klein Hesselink, E. N. *et al*. Effects of radioiodine treatment on salivary gland function in patients with differentiated thyroid carcinoma: a prospective study. *J. Nucl. Med*. 1–29, 10.2967/jnumed.115.169888 (2016). 10.2967/jnumed.115.169888 27339871

[14] Jellema AP, Slotman BJ, Doornaert P, Leemans CR, Langendijk JA (2007). Impact of radiation-induced xerostomia on quality of life after primary radiotherapy among patients with head and neck cancer. Int. J. Radiat. Oncol. Biol. Phys..

[15] Herrmann G, Müller K, Behr M, Hahnel S (2017). Xerostomie und ihr Einfluss auf die mundgesundheitsbezogene Lebensqualität. Zeitschrift fur Gerontologie und Geriatrie.

[16] Jonsson R, Moen K, Vestrheim D, Szodoray P (2002). Current issues in Sjögren’s syndrome. Oral Dis..

[17] Al-hashimi I (2005). Xerostomia Secondary to Sjögren’s Syndrome in the Elderly. Drugs Aging.

[18] Femiano F (2011). A comparison of salivary substitutes versus a natural sialogogue (citric acid) in patients complaining of dry mouth as an adverse drug reaction: A clinical, randomized controlled study. *Oral Surgery*. Oral Med. Oral Pathol. Oral Radiol. Endodontology.

[19] Ship JA, McCutcheon JA, Spivakovsky S, Kerr AR (2007). Safety and effectiveness of topical dry mouth products containing olive oil, betaine, and xylitol in reducing xerostomia for polypharmacy-induced dry mouth. J. Oral Rehabil..

[20] Gil-Montoya JA, Guardia-López I, González-Moles MA (2008). Evaluation of the clinical efficacy of a mouthwash and oral gel containing the antimicrobial proteins lactoperoxidase, lysozyme and lactoferrin in elderly patients with dry mouth–a pilot study. Gerodontology.

[21] Oh D-J, Lee J-Y, Kim Y-K, Kho H-S (2008). Effects of carboxymethylcellulose (CMC)-based artificial saliva in patients with xerostomia. Int. J. Oral Maxillofac. Surg..

[22] Van der Reijden WA, Veerman ECI, Van Nieuw Amerongen A (1994). Rheological properties of commercially available polysaccharides with potential use in saliva substitutes. tID Pergamon Biorheol..

[23] Furness S, Worthington HV, Bryan G, Birchenough S, McMillan R (2011). Interventions for the management of dry mouth: topical therapies. Cochrane database Syst. Rev..

[24] Charnley J (1960). The lubrication of animal joints in relation to surgical reconstruction by arthroplasty. Ann. Rheum. Dis..

[25] Bongaerts JHH, Rossetti D, Stokes JR (2007). The lubricating properties of human whole saliva. Tribol. Lett..

[26] Bongaerts JHH, Cooper-White JJ, Stokes JR (2009). Low biofouling chitosan-hyaluronic acid multilayers with ultra-low friction coefficients. Biomacromolecules.

[27] Wang X, Du M, Han H, Song Y, Zheng Q (2015). Boundary lubrication by associative mucin. Langmuir.

[28] Stokes JR, Boehm MW, Baier SK (2013). Oral processing, texture and mouthfeel: From rheology to tribology and beyond. Current Opinion in Colloid and Interface Science.

[29] Pradal C, Stokes JR (2016). Oral tribology: Bridging the gap between physical measurements and sensory experience. Curr. Opin. Food Sci..

[30] Skedung L (2011). Tactile perception: Finger friction, surface roughness and perceived coarseness. Tribol. Int..

[31] Ranc H (2006). Friction coefficient and wettability of oral mucosal tissue: Changes induced by a salivary layer. Colloids Surfaces A Physicochem. Eng. Asp..

[32] Vissink, A., Wolff, A. & Veerman, E. C. I. In *Salivary**diagnostics* (ed Wong, D. T.) 37–59 (Wiley-Blackwell, 2008).

[33] Prinz JF, de Wijk RA, Huntjens L (2007). Load dependency of the coefficient of friction of oral mucosa. Food Hydrocoll..

[34] Vardhanabhuti B, Cox PW, Norton IT, Foegeding EA (2011). Lubricating properties of human whole saliva as affected by ß-lactoglobulin. Food Hydrocoll..

[35] Obreque-Slier E, Espínola-Espínola V, López-Solís R (2016). Wine pH Prevails over Buffering Capacity of Human Saliva. J. Agric. Food Chem..

[36] Brossard N, Cai H, Osorio F, Bordeu E, Chen J (2016). ‘Oral’ Tribological Study on the Astringency Sensation of Red Wines. J. Texture Stud..

[37] Olsson H, Axéll T (1991). Objective and subjective efficacy of saliva substitutes containing mucin and carboxymethylcellulose. Scand. J. Dent. Res..

[38] Vissink A, De Jong HP, Busscher HJ, Arends J, s-Gravenmade EJ (1986). Wetting properties of human saliva and saliva substitutes. J. Dent. Res..

[39] Tabak LA, Levine MJ, Mandel ID, Ellison SA (1982). Role of salivary mucins in the protection of the oral cavity. J. Oral Pathol. Med..

[40] Levine MJ (1987). Structural aspects of salivary glycoproteins. J. Dent. Res..

[41] Veeregowda DH (2012). Role of structure and glycosylation of adsorbed protein films in biolubrication. PLoS One.

[42] Wu AM, Csako G, Herp A (1994). Structure, biosynthesis, and function of salivary mucins. Mol. Cell. Biochem..

[43] Edgar WM (1992). Saliva: its secretion, composition and functions. Br. Dent. J..

[44] Carpenter GH (2013). The secretion, components, and properties of saliva. Annu. Rev. Food Sci. Technol..

[45] Schipper RG, Silletti E, Vingerhoeds MH (2007). Saliva as research material: Biochemical, physicochemical and practical aspects. Arch. Oral Biol..

[46] Mohamed, R., Campbell, J.-L., Cooper-White, J., Dimeski, G. & Punyadeera, C. The impact of saliva collection and processing methods on CRP, IgE, and Myoglobin immunoassays. *Clin. Transl. Med*. **1** (2012). 10.1186/2001-1326-1-19 PMC3560976 23369566

[47] Madsen JB (2016). Structural and mechanical properties of thin films of bovine submaxillary mucin versus porcine gastric mucin on a hydrophobic surface in aqueous solutions. Langmuir.

[48] Vissink A (1983). A clinical comparison between commercially available mucin- and CMC-containing saliva substitutes. Int. J. Oral Surg..

[49] Forster H, Fisher J (1996). The influence of loading time and lubricant on the friction of articular cartilage. Proc. Inst. Mech. Eng. part H J. Eng. Med..

[50] Rossetti D, Bongaerts JHH, Wantling E, Stokes JR, Williamson AM (2009). Astringency of tea catechins: More than an oral lubrication tactile percept. Food Hydrocoll..

