# Supplementary material for: An ex vivo salivary lubrication system to mimic xerostomic conditions and to predict the lubricating properties of xerostomia relieving agents
Source: Sci Rep. 2018 Jun 14;8:9087. doi: 10.1038/s41598-018-27380-7 (PMC6002370; doi:10.1038/s41598-018-27380-7)
Supplement: Supplementary file 1 — Supplementary Data [file 41598_2018_27380_MOESM1_ESM.docx]

Supplementary Information

## An *ex vivo* salivary lubrication system to mimic xerostomic conditions and to predict the lubricating properties of xerostomia relieving agents

Jeroen Vinke^1^, Hans J. Kaper^1^, Arjan Vissink^2^, Prashant K. Sharma^1*^

^1^ University of Groningen and University Medical Center Groningen, Department of Biomedical Engineering, Groningen, The Netherlands

^2^ University of Groningen and University Medical Center Groningen, Department of Oral Maxillofacial Surgery, Groningen, The Netherlands

## Materials and Methods

**Tongue-enamel normal force estimation**

The normal forces that have to be applied between bovine enamel against porcine tongue during sliding in the various experiments was estimated from experiments with eight healthy volunteers. The volunteers were asked to feel the roughness of a battery powered (cooking) weighing spoon by gently applying pressure in vertical direction using their tongues. Each volunteer performed this experiment in triplicate. The applied force was measured in grams, and converted to normal forces by multiplying the weight by the gravitational constant (9.8 m/s^2^).

**Pear juice and wine preparation**

Pear juice (PJ) was made from Xenia pears and red wine (RW) from Famille Castel Réserve de France, Cabernet Sauvignon 2013, Pays d’Oc. The pears were peeled and blended to pulp with a kitchen blender. The pear pulp was centrifuged at 10000 rpm (10100 g) at 10 °C for 5 minutes in a clean centrifuge tube. The juice was poured over and stored at ice. Prior to the intake of the lubricants, the participants rinsed their mouth for 15 seconds with tap water.

## Results

**Tongue-enamel normal force estimation**

The average weight measured by the weighing spoon for each healthy volunteer is displayed in Supplementary table S1. The average weight was calculated on 23.7±5.7 g. The average weight was converted in to applied vertical force by simple multiplication of the gravitational constant to get the value of 0.24 N. The Normal load in the UMT-3-device was set at 0.25 N as the machine’s a minimal step range of 0.05 N.

Supplementary Table S1. The average weight measured by the weighing spoon for each volunteer. This experiment was performed in triplicate. A personal average and standard deviation (SD) were calculated as well as a total average from 8 volunteers and error propagated SD.

|  | Measured weight (g) | | | Average ± SD (g) |
| --- | --- | --- | --- | --- |
|  | 1 | 2 | 3 |  |
| Subject 1 | 31 | 20 | 15 | 22.0 ± 6.7 |
| Subject 2 | 27 | 35 | 19 | 27.0 ± 6.5 |
| Subject 3 | 15 | 22 | 16 | 17.7 ± 3.1 |
| Subject 4 | 19 | 18 | 16 | 17.7 ± 1.2 |
| Subject 5 | 24 | 35 | 18 | 25.7 ± 7.0 |
| Subject 6 | 33 | 23 | 26 | 27.3 ± 4.2 |
| Subject 7 | 30 | 18 | 40 | 29.3 ± 9.0 |
| Subject 8 | 25 | 26 | 17 | 22.7 ± 4.0 |
| Total average ± SD | |  |  | 23.7 ± 5.7 |

**Effect of salivary dilution on *ex vivo* Friction**

On the tongue-enamel system 1:1 dilution of stimulated whole saliva (SWS) with demineralized water (DW) caused a significant decrease in Relief from 4.8 ± 1.42 to 2.7 ± 0.43 (Figure S1a). Similar dilution with RW caused a decrease in Relief to 1.8 ± 0.37 which was very similar to the Relief provided by DW alone. Dilution with PJ gave similar Relief as SWS diluted with DW. Saliva dilution with DW and PJ provided Relief for a similar duration of 1.5 minutes similar to DW alone (Figure S1b). Dilution with RW did not significantly decrease the Relief period of 5 minutes provided by SWS.

On the (polydimethylsiloxane) PDMS-PDMS system 1:1 dilution of SWS with DW significantly increased the Relief from 69.7 ± 6.32 to 91.5 ± 9.10 (Figure S1c). Similar dilution with RW or PJ resulted in significant drop in Relief to about 60, with no significant difference between them. Dilution of SWS with DW, RW and PJ did not significantly change the Relief period and it varied between 18 to 25 minutes (Figure S1d).


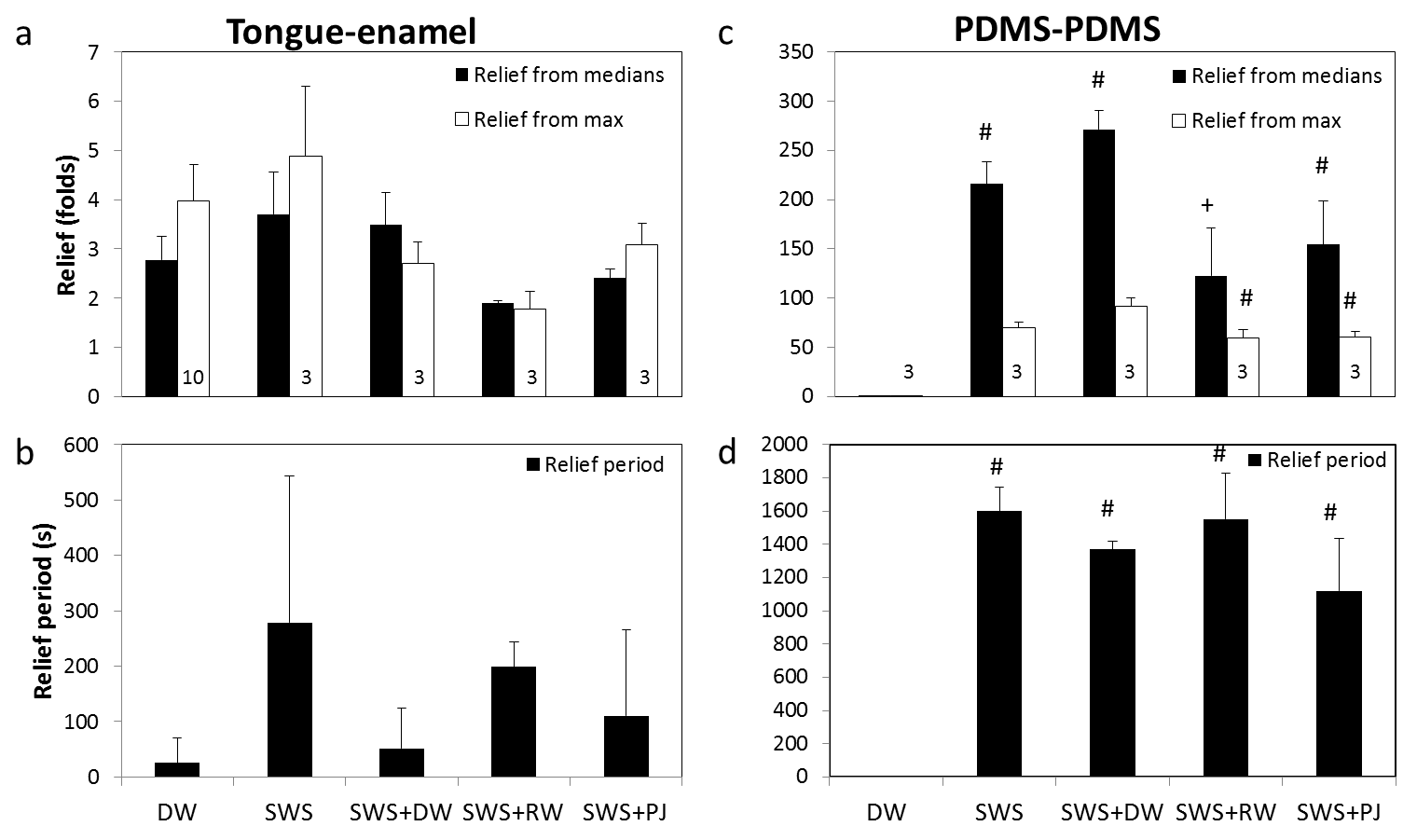


Supplementary figure S1. Mixed saliva lubrication results. *Ex vivo* evaluation of saliva mixtures on the tongue-enamel (a, b) and the PDMS-PDMS (c, d) friction system. Relief calculated based on both *COF_median_* and *COF_max_* for demineralized water (DW), saliva (SWS), and SWS mixtures with DW, red wine (RW) and pear juice (PJ) (a). Relief period in seconds for the same lubricants (b). Relief based on both *COF_median_* and *COF_max_* in the PDMS-PDMS sliding system for DW, SWS and SWS mixtures (c). Relief period in the PDMS-PDMS sliding system (d). ^#^ means statistical difference in respect with demi water, ^+^ means statistical differences with SWS + DW (p <0.05). The number of experiments is mentioned in each column.
